# Evaluating the Quality of Information in Instagram Posts Related to Spine Surgery: A Methodological Study

**DOI:** 10.7759/cureus.72259

**Published:** 2024-10-24

**Authors:** Omer Faruk Sahın

**Affiliations:** 1 Department of Neurosurgery, Ordu University, Ordu, TUR

**Keywords:** data quality, neurosurgery, scoliosis, spinal discectomy, spine

## Abstract

Objective

The purpose of this study is to evaluate the quality of information in Instagram posts related to spine surgeries.

Materials and methods

Seven hashtags related to the most common spine surgeries were identified (#scoliosissurgery, #spondylolisthesis, #microdiscectomy, #spinalfusionsurgery, #spinesurgery, #acdfsurgery, and #vertebroplasty). The first 15 posts listed by the Instagram algorithm for each hashtag were recorded. A total of 105 posts were found, of which 96 were relevant to the research topic, and they were evaluated by two independent physicians using a Modified DISCERN analysis consisting of five questions. Descriptive statistics were calculated, and comparisons were made using the Kruskal-Wallis test and Mann-Whitney U test (p=0.05).

Results

Fifty percent of the posts (n=48) were shared by physicians, and 50% (n=48) were shared by patients. Among these, 79.2% were photos (n=76), and 20.8% were videos (n=20). When evaluating the information quality of patient and physician posts, the information quality of the physician group was found to be significantly higher than that of the patient group (p<0.001). However, there was no significant difference between the information quality of photo and video posts (P=0.129).

Conclusion

Although the posts shared by physicians are considered more reliable than those shared by patients, Instagram posts are not regarded as a reliable source of information for patients.

## Introduction

Social media is increasingly gaining popularity and becoming an essential part of communication [1]. The rise of social media began in the early 2000s with platforms like MySpace and Friendster. While MySpace was the first platform to reach 1 million users, today, YouTube, Instagram, WeChat, Facebook, and Tumblr are the most widely used platforms in the world. A study conducted in the United States found that 59% of Twitter users, 32% of YouTube users, 28% of Instagram users, and 54% of Facebook users regularly receive news from these sites [2]. However, the information available online may not always be accurate or come from reliable sources.

Instagram, a popular social media application that allows users to post photos and videos, has established "hashtags" to facilitate user interaction on specific topics. These hashtags are defined by entering keywords preceded by the hash symbol (#) to indicate relevant topics [3]. This platform not only enables a large number of users to obtain information about diseases and learn from the experiences of other patients but also allows physicians to share their knowledge with patients. However, patients' lack of information and the sharing of experiences can be misleading, potentially leading to misconceptions about the disease and negatively affecting the treatment process. Therefore, it is crucial to quantitatively evaluate the information in these posts.

Certain scales are used for quantitative measurement. Previous studies have utilized scoring systems such as the Health on the Net Foundation Code of Conduct (HON Code) [4], Global Quality Score (GQS), Journal of American Medical Association (JAMA) score [5], Modified DISCERN Scale (Modified DISCERN) [6], and usefulness scoring [7-9].

In previous studies, posts related to orthopedic surgeries, including pediatric scoliosis, anterior cruciate ligament surgery, hip arthroscopy, total joint arthroplasty, and shoulder and elbow surgeries, have been evaluated [10-13]. In the field of spine surgery, the information quality of social media posts related to spinal fusion [1], microdiscectomy [14], and anterior cervical discectomy and fusion (ACDF) [2] has been examined.

The aim of this study is to quantitatively evaluate the information quality of Instagram posts by doctors and patients regarding the most commonly performed procedures in spinal surgery using the Modified DISCERN Scale. The first null hypothesis states that there will be no difference in information quality between the types of users. The second hypothesis is that there will be no significant difference in the format of the posts.

## Materials and methods

Since the data is publicly available, ethical approval is not required for this study. To eliminate biases, a new Instagram account was created. The study data were obtained by two researchers from publicly available Instagram posts recorded using the same Instagram account between August 1 and August 15, 2024. The search tags were determined as the most common spine surgeries: #scoliosissurgery, #spondylolisthesis, #microdiscectomy, #spinalfusionsurgery, #spinesurgery, #acdfsurgery, and #vertebroplasty. The Uniform Resource Locator (URL) of the 15 most popular posts identified by the Instagram search algorithm for each tag was recorded (n=15) (see Appendices). The data obtained from the seven tags were filtered by two researchers using the following exclusion criteria: (1) posts not in English, (2) posts with likes and views disabled, (3) duplicate posts, and (4) posts unrelated to the topic. The relevant posts were then categorized according to predetermined criteria, including user type (patient/doctor) and the format of the post (photo/video).

The quality of information in the Instagram posts was analyzed under five headings using the Modified DISCERN Scale (Modified DISCERN), as shown in Table 1, by two independent evaluators. In cases of disagreement, the posts were evaluated together by the two researchers. The first of these researchers is a 4-year neurosurgery specialist, while the second is an 8-year neurosurgery specialist, and both have gained experience in their professions. The responses to the questions were scored as follows: a "yes" score of 1 and a "no" score of 0, with higher scores indicating greater accuracy and quality.

**Table 1:** The Modified DISCERN Scale

Modified DISCERN
Is the aim clear, concise, and understandable?
Are sources of information reliable? (cited publication, video content was from valid studies)
Is the information presented balanced and unbiased? (any reference to other treatment choices?)
Are additional sources of information listed?
Does it refer to areas of uncertainty?

Statistical analysis was performed using Python software (Version 3.8). The normality of quantitative data was assessed using the Shapiro-Wilk test, revealing non-normal distributions in all seven groups (P<0.05). ). To evaluate the statistical significance of Modifiye DISCERN Score different user groups and formats of posts, Kruskal-Wallis and Mann-Whitney U tests were conducted.

## Results

A total of 146,000 posts were associated with the seven included hashtags, with the highest number of posts under the hashtag #Scoliosissurgery (73,700). This was followed by #spondylolisthesis (45,400), #microdiscectomy (20,300), #acdfsurgery (+1,000), #spinesurgery (+1,000), #vertebroplasty (+1,000), and #spinalfusionsurgery (+500). All 105 posts were in English. Nine posts were excluded for being irrelevant to the topic. No updated images or videos were present. The analysis of the 96 posts regarding user type (patient/doctor) and the format of the post (photo/video) is shown in Table 2.

**Table 2:** Posts Analysis Table

	Type of user		Format of Post	
Hashtags	Doctor	Patient	Photograph	Video
#scoliosissurgery	7	8	12	3
#spondylolisthesis	5	4	7	3
#microdiscectomy	3	11	12	2
#acdfsurgery	7	6	11	2
#spinesurgery	11	3	11	3
#vertebroplasty	13	2	11	3
#spinalfusionsurgery	2	14	12	2

For the Modified DISCERN Scale, the Shapiro-Wilk normality test was conducted to determine the normal distribution of the quantitative data. According to the test results, the groups did not exhibit a normal distribution (p < 0.05). Subsequently, the Mann-Whitney U analysis was performed to assess the statistical significance between the groups. In the evaluation comparing patients and physicians, the information quality of the physician group was found to be significantly higher than that of the patient group (p < 0.001). There was no significant difference in the quality of information between photo and video posts (p = 0.129) (Table 3).

**Table 3:** The mean, standard deviation values and p-value Mann-Whitney U analysis was performed to assess the statistical significance between the groups.

	Modified DISCERN Score (Mean ±SD)	p	Test Statistic
Doctor	2.64 ± 1.52	p < 0.001	1912.0
Patient	0.53± 0.75		1912.0
Photo	1.43±1.58	p = 0.129	597.0
Video	2± 1.56		597.0

## Discussion

This study aimed to quantitatively evaluate different sources of information available to patients by assessing the quality of information in Instagram posts related to spine surgeries. The data obtained indicated that the quality of information in physician posts was higher than that in patient posts, leading to the rejection of the first hypothesis. When evaluated based on the type of posts, it was found that there was no difference in the quality of information between photo and video posts, thus supporting the second hypothesis.

Before the digital age, patients would have preoperative or postoperative discussions either in hospitals or verbally with friends and family. Today, social media brings together patients and doctors through shared treatment options and preoperative and postoperative experiences.

Recent studies have focused on evaluating the quality of social media posts. Aksoy et al assessed YouTube videos related to defibrillation practices in cardiopulmonary resuscitation [15]. Cui et al evaluated the quality of information in TikTok posts regarding mitral valve insufficiency [16]. Given Instagram's emergence as a trending platform among the younger population, the reliability and usability of Instagram posts are frequently evaluated [17,18]. In this study, the quality of information in Instagram posts was assessed using the Modified DISCERN scoring system, as done in previous studies [18,19]. The "Top 15 posts" associated with each hashtag were screened, taking into account patients' search strategies [17,18]. Şahi̇n and her colleagues analyzed the results of their study on smile aesthetics in social media using the mDİSCERN scoring system [20]. In this study, the quality of knowledge of physicians was significantly higher than that of patients and dental techniques. Korkmaz and his colleagues analyzed the information quality of YouTube videos on Unilateral Biportal Endoscopic Spine Surgery using the mDISCERN scoring system [21]. The analysis revealed that although the information quality varies by country, YouTube videos are of high quality and reliability.

There are studies evaluating social media posts related to spine surgeries. Roumeliotis et al examined perceptions related to lumbar microsurgery [14], while Holderread and colleagues analyzed social media posts related to patients who underwent "ACDF" (anterior cervical discectomy and fusion) [2]. Rizkalla and colleagues conducted a similar study focusing on spinal fusion [1]. All three studies centered on patients' surgical satisfaction in social media posts. In this study, the reliability of information in posts related to the most common spine surgical procedures was evaluated at both the patient and physician levels using the Modified DISCERN test. This study is among the first to evaluate the quality of information in Instagram posts related to spine surgeries using the Modified DISCERN scoring system.

In many previous studies, the quality and accuracy of information in patient and physician posts were compared, and it was found that the quality of information in physician posts was higher, aligning with the results of this study [15,18].

Our study will guide future studies by examining Instagram posts more than once a year, creating a larger database, and also examining the data evaluated in the study on other social media networks (YouTube, TikTok, Twitter).

Limitations

This study had some significant limitations. One limitation is that while Instagram is a dynamic platform, the analysis of hashtags and posts was conducted over a specific date range. This study is limited to publicly available posts, and data related to private, deleted, and time-limited posts (such as stories) are missing. In future studies, analyzing Instagram posts at various time points throughout the year would be beneficial. Our study was conducted only on Instagram. Other social media platforms were not used. It was also conducted using a single scoring system. In future studies, multiple scoring systems can be used and evaluated on different social media platforms. Another limitation is that only English posts were considered. Posts regarding spinal diseases and surgeries could be re-evaluated through multinational studies.

## Conclusions

Social media influences patients' decision-making processes regarding medical treatments. Therefore, patients should be cautious when researching medical procedures on social media platforms. Physicians should be more active on social media, making more posts and supporting their information with evidence-based content.

## Appendices

List of URLs used in the study

#Spinesurgery

https://www.instagram.com/reel/C7tVmdkAk90/?igsh=MTBzcHp5bHh5cTQ5eg==

https://www.instagram.com/reel/C5KGKcEujWS/?igsh=MTB2d2cwcm0zMjR5aw==

https://www.instagram.com/reel/C8k5AAzgo7t/?igsh=MWVsd2F3dDN2bDVneQ==

https://www.instagram.com/p/C6omgFCIZ8V/?igsh=aDR2YzIxbDFyam8x

https://www.instagram.com/p/C9NQCp_pERI/?igsh=aWltc3IzNXBhamwz

https://www.instagram.com/p/C9eR5kTupQs/?igsh=MTJubHkzaHRxbXZmNA==

https://www.instagram.com/p/C7drywnv1R8/?igsh=dGxxeHlpaXJha2F0

https://www.instagram.com/p/C6RLB36LAul/?igsh=MTZqMGV5eXl4bmliYg==

https://www.instagram.com/p/C8nu93huHno/?igsh=ODJxOHZwM2N4Z2Nz

https://www.instagram.com/p/CNIDIWfDQhb/?igsh=dzZwanVrem00Ynlk

https://www.instagram.com/p/C5WDvB5RsiQ/?igsh=YzdtdDA2OHlhN3Z3

https://www.instagram.com/p/C9pkw0gp3qq/?igsh=aGJqNW5obW45OHJl

https://www.instagram.com/p/C9mA6S2Iqyy/?igsh=emdxeWY2bWxqMzRn

https://www.instagram.com/p/C8aDKLltzzC/?igsh=bTRxNnBjdGdobHJp

https://www.instagram.com/p/C9lKVD-SXGn/?igsh=bGJwc2ZpenI5b3Ni

#Mikrodiscetomy

https://www.instagram.com/reel/C2CIJqCr9Mt/?igsh=MWZ6NjA5NDZpOWtrcw==

https://www.instagram.com/reel/C9LoH0ISb6O/?igsh=cnYwb2UxZXpvZXJo

https://www.instagram.com/reel/C7WjTL-iNoz/?igsh=N3JuNm95NmFweDRz

https://www.instagram.com/reel/CnSbv1Oggdy/?igsh=MXczcGRoNWFpcWV6Ng==

https://www.instagram.com/p/C2CU7SiPV6U/?igsh=a2ozYmhxZ2V1Y2Q4

https://www.instagram.com/p/CGoJtwdJMln/?igsh=MWplemsxZ2tmdWVqaw==

https://www.instagram.com/p/BeCLVNAnYhQ/?igsh=dnF1OXE1MjJhY3A0

https://www.instagram.com/p/CE1508tAtPG/?igsh=cWJzc2IzdjF6M2Js

https://www.instagram.com/p/BqIJzleAErs/?igsh=MThpeTNmeWk4Ymg3aA==

https://www.instagram.com/p/C5FsQNkr7ZS/?igsh=MW54YmQ3ZDN2cWNhag==

https://www.instagram.com/p/BtyT4sBgAsq/?igsh=MXQwMWYyb3pvc3Aybg==

https://www.instagram.com/p/Bsdx2eJFpT4/?igsh=ejlkd3g4NW55Mnc1

https://www.instagram.com/p/CYSD4mhondd/?igsh=MXl1YTQweWdobHF0

https://www.instagram.com/p/CkiguqOO8nK/?igsh=MWlzcm1ocmdtNDVydg==

https://www.instagram.com/p/CNdBLwHAtUY/?igsh=MTV2czMzOWVpM3ZqZA==

#acdfsurgery

https://www.instagram.com/reel/CqVIf7iPiZe/?igsh=MXFoOThuZ2s0YTg2cQ==

https://www.instagram.com/p/C9exNJAtfos/?igsh=MWhxM285eWt2Z3RmMg==

https://www.instagram.com/p/CV-tD42hMTi/?igsh=ZTFpYXJqczU0NzB4

https://www.instagram.com/p/CE5LPJShS5Q/?igsh=MWNvdno2aWY1NGp3cg==

https://www.instagram.com/p/CNBA4wCl45X/?igsh=MTQxYW1sMWd0N2tmaw==

https://www.instagram.com/p/CTeGeYMK0H-/?igsh=cnpicTd0MHVnM2Q3

https://www.instagram.com/p/CZI613rMDXh/?igsh=NGd6cmNjNDZhY2Jw

https://www.instagram.com/p/CMjZTNDh5c2/?igsh=MWN5b2p1Z2RpeGxubw==

https://www.instagram.com/p/C9dzYdXPCur/?igsh=cjJlOXUwMTM1cGw3

https://www.instagram.com/reel/Ca9kbo1lUSs/?igsh=MWRiejhtZnVtZjZoYg==

https://www.instagram.com/reel/C7kUzCNP1p8/?igsh=MWNnMDNqMGw5N3NzYg==

https://www.instagram.com/p/CekKKPLoZDu/?igsh=MWwxdGwycmU2bHF4dQ==

https://www.instagram.com/p/Bxyd4FVBIT5/?igsh=MXZpa3Bmb2lqbXp4MQ==

https://www.instagram.com/p/CvGn3UHuULm/?igsh=cWd2MDRmY2V0ZGhv

https://www.instagram.com/p/C2chDIKMZ7C/?igsh=cmd4ODRhMmdhZTh2

#scoliosissurgery

https://www.instagram.com/p/CRHMT5Hs76s/?igsh=MTJ3cmQzZnpjZXh5OQ==

https://www.instagram.com/p/C7rQCu_OqZl/?igsh=MXR6ODlmdGxubno3Yw==

https://www.instagram.com/p/B8qvj6PBJVR/?igsh=YXV5OGF4d3A4MG1t

https://www.instagram.com/reel/CtPaqLzgErF/?igsh=MWU4aXlkbHlwbnkwNA==

https://www.instagram.com/reel/CpOQLdzAq4b/?igsh=MWwweHF1M3FmeGFkMQ==

https://www.instagram.com/p/C9y8PjcoeLD/?igsh=cXg2emhrZmxjMWFw

https://www.instagram.com/p/CIN4ZoyAfO3/?igsh=cjN2bTl0dWFwdmsw

https://www.instagram.com/p/CgMxxTmJwLJ/?igsh=NWcyaHY1djhhYWNr

https://www.instagram.com/p/C8VfRYHO-e5/?igsh=ODlxaHoybTNwa3E3

https://www.instagram.com/p/C4094ktSRyp/?igsh=eGQ3YTlmZXJoaDln

https://www.instagram.com/p/Bz-yJ_Qnbw0/?igsh=cWJ3bHNvcndhNzYz

https://www.instagram.com/p/CfR9FS0AIgK/?igsh=OWY1c2FmOTlpczUy

https://www.instagram.com/p/C9ne8mMRpq3/?igsh=MXU0cWF5NWY1Zm14cQ==

https://www.instagram.com/p/C8sFx-VhpJS/?igsh=MXIxNWlpanU5aTAwZQ==

https://www.instagram.com/p/ByqRuQ-g50R/?igsh=czZubGl5eW8zaDB6

#Spinalfusionsurgery

https://www.instagram.com/reel/CrgLqgAIkrq/?igsh=cWVseTg5aTF5em04

https://www.instagram.com/reel/C0EyQsooiCx/?igsh=aDRxa3d0OWVlazFi

https://www.instagram.com/reel/Cs_uZ-KArEi/?igsh=cGQ5MmgyYnJic2U0

https://www.instagram.com/p/C9m540Lqt_Y/?igsh=MWVvNzNwZHp0cGk5aQ==

https://www.instagram.com/p/C9TXtVDtXPU/?igsh=ODFsNnQ1YXZpcHBz

https://www.instagram.com/p/BhIHEdyD5H1/?igsh=cXZtdTg5bnZ1d281

https://www.instagram.com/p/C9iER48vq6I/?igsh=MTRtbDdkbW9lNzgxbw==

https://www.instagram.com/p/C32vzhhrwjf/?igsh=NG92YWhucWdwbDRl

https://www.instagram.com/p/Cm_bgU9Be6U/?igsh=MXc0ZnR1a3djcXgxYg==

https://www.instagram.com/p/CChirsrjYiP/?igsh=ZXlic2lnZ3E5NWpn

https://www.instagram.com/p/CegT7fErw_L/?igsh=MWlnZm9yNWNqeDh1Ng==

https://www.instagram.com/p/CGi6K6TDwZ0/?igsh=MXBibDNqM3Q4cmM4YQ==

https://www.instagram.com/p/CxbTVzjsIAW/?igsh=MW4yaW45MHhzOWRuMQ==

https://www.instagram.com/p/C48b6YZOUth/?igsh=YzRwZG1scjY0ZmNp

https://www.instagram.com/p/Cg2LU3WJocN/?igsh=MW1tajNyenQ5dHo0bA==

#vertebroplasty

https://www.instagram.com/reel/C2Gnq3-xirx/?igsh=MXVmaGZubXJ5YnB4OQ==

https://www.instagram.com/reel/CnsFPqtoY_w/?igsh=dW1wZnljZDU3bG53

https://www.instagram.com/reel/CVgfXaFFSY9/?igsh=bGs2b3B2bjN2eWdh

https://www.instagram.com/p/CqGUro4hwyj/?igsh=YnFyaThiNTA0Y25r

https://www.instagram.com/p/CdqW8-BMEBH/?igsh=MXN1aTdvdGRobnBrYQ==

https://www.instagram.com/p/CaBmzX9Pujn/?igsh=MXNsYTFlMWowbGswZg==

https://www.instagram.com/p/C3bx0-KI2-D/?igsh=MTRuNTFkd2lqcjQ5MA==

https://www.instagram.com/p/C2DGlJ7iAAY/?igsh=aGdidHYydXV5YWN5

https://www.instagram.com/p/CiomchPtHAL/?igsh=ODEzdjgyemM3Z21l

https://www.instagram.com/p/C1wGrwUvbVY/?igsh=MTN4cmdsYmVnYm5idg==

https://www.instagram.com/p/C2tPWHLCK2w/?igsh=MWxiYmI2d3l2ZjU4eA==

https://www.instagram.com/p/B9EHDNLihOX/?igsh=Z3Q0Y245YTB1azBl

https://www.instagram.com/p/C6bk4JfvwhO/?igsh=MWJ4cmN2YWVzZndxcA==

https://www.instagram.com/p/CtuNUEqtobc/?igsh=MXR1ZG83ODQzN3J4aA==

https://www.instagram.com/p/CZ6dVQtodEb/?igsh=bDRnMDFzdWdvMndr

#spondylolisthesis

https://www.instagram.com/reel/C1o3wd7pppG/?igsh=dWlhb2x4Z25tZmRo

https://www.instagram.com/reel/CWoyZVvgAMd/?igsh=YnBqcW9jMTlhYzZk

https://www.instagram.com/reel/CeGrsAmKHrk/?igsh=eGNvMHdrYm15enlu

https://www.instagram.com/p/C6LWcGsLCcG/?igsh=Y2xic3BsdDZlbnhy

https://www.instagram.com/p/C5cPGFAJK0u/?igsh=MTVzenF6a2VsYnYyNw==

https://www.instagram.com/p/BsywCz5g8Pn/?igsh=MWpveXUyanI5MGQxbQ==

https://www.instagram.com/p/C7MLklFPg49/?igsh=dzhwMGYxcGR4cmxm

https://www.instagram.com/p/C62VBwLP3nc/?igsh=b3BlM3F3ZnJ6a3A4

https://www.instagram.com/p/CWU-fY7ssE2/?igsh=MWh2bTl3OHcwM2J0cg==

https://www.instagram.com/p/C6tmGp_qAtB/?igsh=MWxzbnFhZ2MxNHl2OA==

https://www.instagram.com/p/CFebfpHj9yd/?igsh=MWU5ZG9pMzQ5dDl6Zg==

https://www.instagram.com/p/C3arnYkMi1Z/?igsh=MWR1NnhtdXB5Y2tyOQ==

https://www.instagram.com/p/CKL7Zq3nDlQ/?igsh=MW9xc3N2dmNjbGgwcA==

https://www.instagram.com/p/C8iTOCyPg9g/?igsh=ZHB4NmJpZmV2NXR0

https://www.instagram.com/p/CR_5rFVIpD2/?igsh=MTZjeDNyazl3Z25sZw==
